# A Dentist’s Relations with Young Patients

**Published:** 1919-02

**Authors:** C. C. Mann


### A DENTIST'S RELATIONS WITH YOUNG PATIENTS

My office has a view of the Entire Puget Sound. The sunsets are very beautiful from the windows. I doubt if you can go anywhere and find more beautiful pictures from day to day than from the windows of my operating room. It gives the children pleasure to observe these pictures; they comment on them and are pleased with them. The movement of the vessels in the Sound—the manufacturing districts where the great ships are built and launched every day now, is always of great interest to the children. To me, a child who is under my care is as if he were my child. I do not agree with Dr. Suggett that a child should be brought up to your plane. You must study what the child likes and dislikes—his susceptibility under certain conditions, and if you do so you will find he will meet you as near your plane as possible, and your mutual understanding will bring about a much easier situation.

I have children who have been with me some years, and some only a short time, and I want to tell you that I have more friends among the little folks that I really care for than I have among the older people. I can depend upon the word of those children just as implicitly as I can depend on the word of my own boy, and so it has been a great pleasure to me to take up this subject as a matter of thought and consideration. I have studied it carefully, and have found the co-operation afforded me by my patients has been a wonderful thing.

If a child comes to me with an unclean mouth, the first thing he does is to apologize for the condition of his mouth, and the children will do so in ninety-nine cases out of a hundred. There is little trouble after once having instructed the patient as to prophylaxis, to secure co-operation.

I think the educational feature of our relations to patients, is a wonderful thing. I think the incorporation of some such idea in the educational institutions will give the young men going out into the profession a better understanding as to these considerations than they can obtain in any other way. Too many enter dentistry with the idea that their dignity should be the first consideration and that it should not be overstepped by anybody. It is beneficial when properly applied, and disastrous when improperly applied. It is easy enough if you have yourself well in hand, well poised, with flexible minds, to be dignified in all cases requiring dignity, and otherwise it may not be.—Dr. C. C. Mann, in *Pacific Gazette.*

